# Chimeric antigen receptor-T-cell therapy: China leading the way

**DOI:** 10.1097/BS9.0000000000000118

**Published:** 2022-07-20

**Authors:** Robert Peter Gale

**Affiliations:** a Imperial College of Science, Technology and Medicine, London, UK

Clinical trials of chimeric antigen receptor (CAR)-T-cell therapy in China far outnumber those in other countries including the United States. A recent Boolean search on ClinicalTrials.gov for *CAR-T-cells* AND *leukaemia* identified 188 studies compared 99 studies in the United States. In lymphoma, there are 234 studies in China versus 159 in the United States and in plasma cell myeloma, 64 versus 55.

There are many reasons for these disparities. One is the speed at which a new study is approved in China versus the United States. Also, there are many more physician/scientists and people needing therapy in China compared with the United States. Many trials in China use CAR-T-cells produced locally at a medical center or in collaboration with a small biotechnology company. This is uncommon in the United States where the requirement for CAR-T-cell approval by the US Food and Drug Administration (US FDA) requires large, complex, expensive clinical trials. There is no way for medical centers or drug companies to recover costs of developing a CAR-T-cell therapy without US FDA approval. This seems less an obstacle in China. Other considerations are too complex to consider here.

Despite the considerable CAR-T-cell therapy clinical research in China only 2 therapies, axicabtagene ciloleucel (Yescarta: Kite, Santa Monica, California) and relmacabtagene autoleucel (Carteyva: JW Therapeutics, Shanghai, China) are approved in China by the National Medical Products Administration (NMPA). Axicabtagene ciloleucel was developed in the United States by a large pharma company (Kite/Gilead) and the NMPA approval followed the US FDA approval by 3.5 years. Relmacabtagene autoleucel was approved by NMPA in September, 2021. It was developed by JW Therapeutics based on a CAR-T-cell process platform of Juno Therapeutics/Bristol Myers Squibb, another US company.

There are many unanswered questions regarding CAR-T-cell therapy such as: Are CAT-T-cells effective in persons the B-cell acute lymphoblastic leukemia (ALL) with central nervous system (CNS) leukemia? and Durability of response to CAR-T-cell therapy in PCM. The wealth of CAR-T-cell therapy clinical trials in China has allowed for some unique contributions. I discuss 2 recent reports from clinical trials groups led by Prof. Kailin Xu and his colleagues from Xuzhou and other centers in China.

In their first article,^1^ the authors studied safety and efficacy of anti-CD19 CAR-T-cells in persons with advanced B-cell acute lymphoblastic leukemia (ALL) with synchronous central nervous system (CNS) leukaemia (NCT02782351 and ChiCTR-OPN-16008526) (1). These persons are typically excluded from registration trials because of safety and efficacy concerns. This phase-2 study had 48 children and adults who received anti-CD19 CAR-T-cells with or without anti-CD22 CAR-T-cells. The bone marrow overall response rate (ORR) was 88% (95% confidence interval [CI], 75, 94%) and the CNS ORR was 85% (73, 93%) in the CNS. Median event-free survival (EFS) was 9 months (4, 19 months) and median survival, 16 months (14, 20 months). 1-year cumulative incidences of relapse (CIR) by sites were 31% and 11%. Nine subjects had grade ≥3 cytokine release syndrome (CRS) and 11, grade ≥3 immune effector cell neurotoxicity syndrome (ICANS). These data expand our knowledge where CAR-T-cell therapy is safe and effective in children and adults with CD19-positive advanced ALL with synchronous CNS leukemia.

In the second study,^2^ Prof. Xu and colleagues report long-term (median 21 months) of safety and efficacy of therapy of combined anti-B-cell maturation antigen (BCMA) and anti-CD-19 CAR-T-cells in 62 evaluable subjects with advanced PCM. The ORR was 92% (82, 97%) and complete response, 60%. 43 subjects were measurable residual disease (MRD)-negative. Estimated median duration of response was 20 months (9, 32 months) estimated median progression-free survival (PFS) and 18 months (10, 27 months). Six subjects had grade ≥ 3 CRS and 2 grade ≥ 3 ICANS. Late adverse events were rare save for B-cell aplasia, hypogammaglobulinemia, and infections. The authors concluded that combined anti-BCMA and anti-CD19 CAR-T-cell therapy is safe and effective in persons with advanced PCM.

These articles are valuable contributions to our rapidly expanding knowledge of safety and efficacy of CAR-T-cell therapy in hematological cancers. Prof. Xu and his colleagues are to be congratulated for helping us along the way.

## ACKNOWLEDGMENTS

RPG acknowledges support from the National Institute of Health Research (NIHR) Biomedical Research Centre funding scheme.

## References

[1] QiYZhaoMHuY. Efficacy and safety of CD19-specific CAR T cell-based therapy in B-cell acute lymphoblastic leukemia patients with CNSL. Blood 2022:139(23):3376–3386.3533877310.1182/blood.2021013733

[2] Wang Y Cao J Gu W. Long-term follow-up of combination of B-cell maturation antigen and CD19 chimeric antigen receptor T Cells in multiple myeloma. J Clin Oncol 2022:40(20):2246–2256. 35333600 10.1200/JCO.21.01676

